# Phenolic diterpenes from Rosemary supercritical extract inhibit non-small cell lung cancer lipid metabolism and synergise with therapeutic drugs in the clinic

**DOI:** 10.3389/fonc.2022.1046369

**Published:** 2022-11-09

**Authors:** Adrián Bouzas, Marta Gómez de Cedrón, Gonzalo Colmenarejo, José Moisés Laparra-Llopis, Juan Moreno-Rubio, Juan José Montoya, Guillermo Reglero, Enrique Casado, Beatriz Tabares, María Sereno, Ana Ramírez de Molina

**Affiliations:** ^1^ Precision Nutrition and Cancer Program, Molecular Oncology Group, IMDEA Food Institute, CEI UAM, CSIC, Madrid, Spain; ^2^ CANAAN Research & Investment Group, Madrid, Spain; ^3^ Biostatistics and Bioinformatics Unit, IMDEA Food Institute, CEI UAM, CSIC, Madrid, Spain; ^4^ Molecular Immuno-Nutrition Group, IMDEA-Food Institute, CEI UAM, CSIC, Madrid, Spain; ^5^ Medical Oncology Department, Infanta Sofía University Hospital, San Sebastián de los Reyes, Madrid, Spain; ^6^ Faculty of Medicine, Complutense University of Madrid, Madrid, Spain; ^7^ Department of Production and Characterization of Novel Foods, Institute of Food Science Research (CIAL) (CSIC.UAM), Madrid, Spain

**Keywords:** lipid metabolism, precision nutrition, NSCLC, rosemary extract, phenolic diterpenes

## Abstract

Lung cancer is one of the most deadly and common cancers in the world. The molecular features of patient’s tumours dictate the different therapeutic decisions, which combines targeted therapy, chemotherapy, and immunotherapy. Altered cellular metabolism is one of the hallmarks of cancer. Tumour cells reprogram their metabolism to adapt to their novel requirements of growth, proliferation, and survival. Together with the Warburg effect, the role of lipid metabolism alterations in cancer development and prognosis has been highlighted. Several lipid related genes have been shown to promote transformation and progression of cancer cells and have been proposed as biomarkers for prognosis. Nevertheless, the exact mechanisms of the regulation of lipid metabolism and the biological consequences in non-small cell lung cancer (NSCLC) have not been elucidated yet. There is an urgent necessity to develop multidisciplinary and complementary strategies to improve NSCLC patients´ well-being and treatment response. Nutrients can directly affect fundamental cellular processes and some diet-derived ingredients, bioactive natural compounds and natural extracts have been shown to inhibit the tumour growth in preclinical and clinical trials. Previously, we described a supercritical extract of rosemary (SFRE) (12 - 16% composition of phenolic diterpenes carnosic acid and carnosol) as a potential antitumoral agent in colon and breast cancer due to its effects on the inhibition of lipid metabolism and DNA synthesis, and in the reduction of resistance to 5-FluoroUracil (5-FU). Herein, we demonstrate SFRE inhibits NSCLC cell bioenergetics identifying several lipid metabolism implicated targets. Moreover, SFRE synergises with standard therapeutic drugs used in the clinic, such as cisplatin, pemetrexed and pembrolizumab to inhibit of cell viability of NSCLC cells. Importantly, the clinical relevance of SFRE as a complement in the treatment of NSCLC patients is suggested based on the results of a pilot clinical trial where SFRE formulated with bioactive lipids (PCT/ES2017/070263) diminishes metabolic and inflammatory targets in peripheral-blood mononuclear cells (PBMC), such as *MAPK (p=0.04)*, *NLRP3 (p=0.044)*, and *SREBF1 (p=0.047)*, which may augment the immune antitumour function. Based on these results, SFRE merits further investigation as a co-adjuvant in the treatment of NSCLC.

**Clinical trial registration:**

ClinicalTrials.gov Identifier NCT05080920

## 1 Introduction

Lung cancer is one of the most deadly and common types of cancer in the world (1). Based on cell origin, about 80–85% of lung cancers are of non-small-cell lung cancer (NSCLC). In the era of genomic medicine, precision oncology has improved the treatment and quality of life of patients (2). For example, in lung adenocarcinomas, the identification of targetable molecular pathways has improved the treatment and outcome of patients. Epidermal growth factor receptor (EGFR) is a key tumour driver and one of the main targets in the successful treatment of NSCLC (3, 4). In addition to EGFR inhibitors -first (erlotinib, gefitinib), second (afatrinib, dacomitinib) or third generation (Osimertinib) inhibitors-, other pathways like ALK or ROS-1 translocations, BRAF, K RAS, RET or NTRK/ROS1 are relevant in the NSCLC landscape (5). Nevertheless, majority of tumours develop drug resistance and a new generation of targeted therapeutic drugs, alone and/or in combination with different drugs, are being developed. Recently, immune checkpoint system inhibitors, such as the anti-PD-1/PD-L1 and anti-CTLA-4 antibodies, have shown additional clinical benefits in combination with chemotherapy (6). In this sense, numerous works indicate that activated EGFR signalling increases the expression of PD-L1, which is also associated to acquired resistance to EGFR-TKIs (7).

The altered cellular metabolism is one of the hallmarks of cancer (8). Tumour cells adapt their metabolism to to support cell growth, proliferation, and survival. In the past years, the relevance of lipid metabolism alterations in cancer has been highlighted (9). Several lipid-related genes in cancer promote transformation and progression and some of the have been proposed as biomarkers for prognosis in cancer (9–11). Lipids are structural components of cellular membranes, provide energy, and are key essential players in the control of the inflammation and oxidative stress (12, 13). Tumour cells present high avidity for fatty acids and cholesterol, which can be satisfied by the activation of *de novo* lipogenesis and cholesterogenesis and, alternatively, by augmenting the uptake of exogenous lipids and/or lipoproteins (14). Nevertheless, the exact mechanisms of regulation and the biological consequences of lipid metabolism in NSCLC have not been elucidated yet.

Nutrients affect fundamental metabolic cellular processes and some diet-derived ingredients, including bioactive natural compounds and natural extracts have been shown to inhibit the tumour growth in preclinical and clinical trials. The development of powerful “omics” technologies has opened new avenues towards nutritional sciences. In this way, genomic, transcriptomic, proteomic, metabolomic, lipidomic and metagenomic analysis have led to a new vision of the delivery of nutritional advice by mean of *Precision nutrition* (15). Previously, we have described a supercritical extract of rosemary SFRE (approved for human use by the European Food Safety Authority-EFSA) as a potential antitumoral agent in colon and breast cancer due to its effects on lipid metabolism, DNA synthesis, and in alleviating the appearance of resistance to 5-FluoroUracil (5-FU) (16–19).

Herein, we demonstrate the inhibitory effects of SFRE in NSCLC cells, alone and in combination with standard chemotherapeutic drugs such as cisplatin, pemetrexed and pembrolizumab. Importantly, we also demonstrate SFRE targets lipid metabolism which seems to be on the bases of the inhibition of NSCLC cell proliferation. In collaboration with the Medical Oncology Department of Hospital Infanta Sofia (Madrid, Spain), we have conducted a pilot clinical trial with NSCLC patients where SFRE formulated with bioactive lipids (PCT/ES2017/070263) modulates metabolic and inflammatory targets in peripheral-blood mononuclear cells (PBMC) after sixteen weeks of treatment. Based on these results, SFRE merits further investigation as a co-adjuvant in the treatment of NSCLC.

## 2 Materials and methods

### 2.1 Reagents and cell culture

Human lung adenocarcinoma cell lines, NCI-H1299 (H1299) and NCI-H1975 (H1975) were obtained from the American Type Culture Collection (ATCC). Normal epithelial cells (CCD 841) were used to compare the effect of SFRE on tumour and normal cells. Roswell Park Memorial Institute (RPMI) 1640 Medium supplemented with 2 mM L-glutamine, fetal bovine serum (FBS) and 0.25% trypsin were purchased from Cultek. Cells were grown in RPMI 1640 supplemented with 10% FBS at 37°C in a humidified atmosphere with 5% CO_2_. Dimethyl sulfoxide (DMSO), cisplatin and pemetrexed were purchased from Sigma (Oakville, ON, Canada).

Supercritical Rosemary Extract (Rosemary extract 25 Type No. 027.020) was purchased from Flavex Naturextrakte GmbH (Rehlingen-Siersburg, Germany). Composition of the extract was 12 - 16% composition of phenolic diterpenes carnosic acid and carnosol (calculated as carnosic acid) with > 10% of carnosic acid; total volatile flavour compounds < 4%, water < 1%, residual content of ethanol < 2%, cuticular waxes, as provided from Flavex.

For the analysis of effects of SFRE on cell bioenergetics, Glucosa XF 1 M (Ref: 103577-100) was purchased from Agilent; Oligomycin A (Ref: 75351), FCCP (Ref: C2920), Rotenone (Ref: R88757), Antimycin A (Ref: A8674), and 2-Deoxy-D-glucose (Ref: D8375) were purchased from Merck. Cisplatin (Ref: P4394) and Pemetrexed (Ref: PHR1596) were purchased from Merk. Pembrolizumab was kindly provided by the Medical Oncology Department of Hospital Infanta Sofía.

### 2.2 MTT proliferation assay

Cell proliferation after different treatments was determined using the 3-(4,5-Dimethylthiazol-2-yl)-2,5-Diphenyltetrazolium Bromide (MTT) method. Briefly, cells were seeded (4,000 cells per well) in 96-well plate in quadruplicates and kept in RPMI 1640 media o/n at 37°C in 5% CO_2_. Then cells were treated with several concentrations of SFRE, cisplatin, pemetrexed or pembrolizumab. Cells treated with DMSO were used as controls. After 48 h, MTT solution was added (0.5 mg/mL final concentration) and cells were incubated for 3 h. 200 μL of DMSO were added to each well to dissolve the formazan crystals and absorbance was measured at 560 nm using a scanning spectrophotometer microplate reader (UVM 340 Biochrom). To determine the number of viable cells, the formazan produced by cells from the MTT (thiazolyl blue tetrazolium bromide) solution (50μL at 5 mg/mL in PBS) was solubilized by adding 200 μL DMSO. Then, the absorbance at 560 nm was measured using a scanning spectrophotometer microplate reader (UVM 340 Biochrom). The parameters IC50 (50% cell proliferation inhibition), GI50 (50% growth inhibition), TGI (total growth inhibition), and LC50 (50% cell death) were calculated according to the NIH definitions using a logistic regression (20, 21). The determination of cell proliferation at the beginning of the treatment (time zero) allows to calculate the parameters related to cell proliferation.

### 2.3 Evaluation of the combination indexes of SFRE with cisplatin, pemetrexed or pembrolizumab

To evaluate synergism between SFRE and therapeutic drugs -cisplatin, pemetrexed or pembrolizumab- NSCLC cells were pre-treated (for three hours in the case of pembrolizumab) and/or were concomitantly treated (in the case of cisplatin and pemetrexed), as indicated, with a fixed concentration of SFRE (1/2xIC_50_) in the presence or not of increasing concentrations of the indicated drugs. The MTT assay was used to evaluate the percentage of the inhibition of cell proliferation relative to non-treated cells. The Combination Index was obtained using CalcuSyn software (Biosoft), which was developed based on the median-effects method by Chou and Talalay. Briefly, CI values significantly lower than 1.0 indicates a synergistic effect; CI values higher than 1.0 indicates antagonism; and additivity as CI values equal to 1.0 (22).

### 2.4 Quantitative real-time polymerase chain reaction in NSCLC cells

Both NCI-H1299 (H1299) and NCI-H1975 (H1975) cells were treated with SFRE for 48 hours at different doses based on the values obtained from cell proliferation (1/2xIC_50_, 1xIC_50_, 2xIC_50_). Non-treated cells were kept as controls. Total RNA was extracted with Tri Reagent (Sigma). Then, 1 µg of RNA was reverse transcribed with High-Capacity RNA to cDNA Master Mix system (Life Technologies, Carlsbad, CA, USA). qPCR was performed in the 7900HT Real-Time PCR System (Life Technologies) using VeriQuest SYBR Green qPCR Master Mix (Affymetrix, Santa Clara, CA, USA). **Supplementary Table 1** indicates the list of Taqman Probes and the sequences of the oligos used in the study. The 2^-ΔΔCt^ method was applied to calculate the relative gene expression (23).

### 2.5 Extracellular flux analysis of the extracellular acidification rates and the oxygen consumption rates

Mitochondrial oxidative respiration (Cell MitoStress Test) and aerobic glycolysis (Cell GlycoStress Test) were monitored with the XFe96 Cell Bionalyzer (Seahorse Biosciences, XFe96). Optimal cell density and drugs titration were previously determined. The dependency of the cells on aerobic glycolysis and oxidative phosphorylation was monitored after the sequential addition of modulators of both bioenergetic pathways. Prior to the experiments, cells were pre-treated with different doses of SFRE for 48 h. Non-treated cells were kept as controls.

Before the experiments, same number of cells, previously pre-treated with SFRE, were seeded in XFe-plates and kept in the absence of any treatment to compare the bioenergetic metabolism of the very same number of cells.

For the *GlycoStress assay*, 10,000 cells were plated in an XFe- 96 well-plate and kept 6 h in RPMI 1640 10% FBS to allow the cells to attach. Then, the culture medium was changed to 0.2 mM glutamine XFe-DMEM (5 mM Hepes pH 7.4) to starve the cells for 30 min. First, basal extracellular acidification rate (ECAR) was measured (1–3 measurements). Glucose (10 mM) was added to determine glycolysis; this is the increased ECAR from the basal situation, after the addition of glucose (4–6 measurements). This parameter indicates the capacity of the cells to use glucose. Next, maximal glycolytic capacity was monitored (7–9 measurements) after the addition of oligomycin (1.5 µM), which inhibits the ATP production from the oxidative mitochondrial respiration. Finally, 50 mM DG was added to specifically shut down aerobic glycolysis (10 to 12 measurements). *Glycolysis* was calculated as: (Maximal rate measurement before oligomycin injection: measurement 6) - (Last rate measurement before glucose injection, measurement 3). *Glycolytic capacity*: (Maximum rate measurement after oligomycin injection, measurement 8) - (Last rate measurement before glucose injection, measurement 3). *Glycolytic reserve* (*Glycolytic capacity*) - (*Glycolysis*). Non-glycolytic Acidification: Last rate measurement prior to glucose injection, measurement 3).

For the *MitoStress assay*, 10,000 cells were plated in an XFe- 96 well-plate, and cells were kept for 6 h in RPMI 1640, 10% FBS to allow the cells to attach. Then, the medium was changed to 10 mM glucose, 2 mM glutamine, and 1 mM pyruvate XFe-DMEM (5 mM Hepes, pH7.4) and, then the cells were incubated for 30 min at 37°C without CO_2_. Three different modulators of the mitochondrial respiratory chain were sequentially injected. After basal oxygen consumption rate (OCR) monitorization (1–3 measurements), oligomycin (1.5 μM), which inhibits ATPase, was added to determine the amount of oxygen dedicated to ATP production by mitochondria (3–6 measurements). To determine the maximal respiration rate or spare respiratory capacity, FCCP (carbonyl cyanide-4-(trifluoromethoxy) phenylhydrazone) was added (0.9 μM) to free the gradient of H^+^ from the mitochondrial intermembrane space (7–9 measurements) and thus to activate maximal respiration. Finally, a mix of antimycin A and rotenone (0.5 μM) was added to completely inhibit the mitochondrial respiration (10–12 measurements).

Main parameters extracted from the bioenergetic profile were calculated as: Non-mitochondrial Oxygen Consumption: Minimum rate measurement after Rotenone/antimycin A injection (measurement 12); Basal Respiration Rate: (Last rate measurement before first injection-measurement 3)-(Non-mitochondrial Respiration Rate, measurement 12); Maximal Respiration Rate: (Maximum rate measurement after FCCP injection, measurement 8)-(Non-mitochondrial Respiration, measurement 12)); H^+^Leak: (Minimum rate measurement after Oligomycin injection, measurement 6)-(Non-Mitochondrial Respiration, measurement 12); ATP production: (Last rate measurement before Oligomycin injection, measurement 3)-(Minimum rate measurement after Oligomycin injection, measurement 6).

### 2.6 Measurement of the intracellular ATP content

The relative cellular ATP content was measured by the ATP-based assay CellTiter-Glo Luminescent Cell Proliferation kit (Promega, Madison, WI, USA; Cat # G7571) with modifications from the manufacturer’s protocol. Briefly, after 48h of treatment with SFRE at the indicated doses, 15,000 cells were plated in a 96-well clear bottom black polystyrene plates. Non-treated cells were plated in a similar way. Cells were then maintained for 6 h in complete media, without treatments, to allow attachment of the cells. Then, an equal volume of the single-one-step reagent provided by the kit was added to each well and rocked for 15 minutes at room temperature. Cellular ATP content was measured by a luminescent plate reader.

### 2.7 Flow cytometry analysis

H1299 and H1975 NSCLC cells pre-treated with SFRE were collected in RPMI 1640 media and centrifuged (1200 rpm, 5min). Cells were washed twice with PBS before adding an adequate volume to obtain a 2% (final concentration) paraformaldehyde solution. According to the manufacturer’s instructions, 1 µl of anti-human PDL1 (FITC Mouse Anti-Human CD274 antibody (Cat. No. 558065), and/or 1 µl of BV786 Rat Anti-Human CX3CR1 antibody (Cat. No. 744489) antibodies were added to cell suspensions. After gentle vortex, samples were incubated for 15 min in the dark. For the analysis of SREBP1, Mouse Anti-human SREBP1 was used as primary antibody (BD, 557036), and Goat anti-Mouse IgG (H+L) as secondary Antibody, Alexa Fluor™ Plus 488 (Cat # A-11001).

Cells were then analyzed using the FACS Diva software (BD Biosciences). At least 10,000 events were analysed for each independent sample. Mean values of arbitrary fluorescence unit for 10,000 cells were obtained and expressed as percentage of control.

### 2.8 Quantification of intracellular neutral lipid content and phospholipids

To analyse the effects of SFRE on intracellular neutral lipid content, we pre-treated H1299 and H1975 cells for 48 h at the indicated doses of SFRE. Non-treated cells were kept as controls. Then, viable cells were collected and 5,000 cells were plated in the absence of any treatment for 6 h to allow the cells to attach Intracellular neutral lipid content was measured using Oil Red O staining (24). Briefly, cells were gently washed with ice-cold PBS (pH 7.4) and then fixed with 10% formalin at room temperature (RT) for 1 h. After removal of the formalin, cells were washed with 60% isopropyl alcohol for 5 min and then washed twice with PBS. Wells were let to dry completely before the addition of filtered Oil Red O solution for 30 min at RT. Stained oil droplets were extracted with 100% isopropanol for 10 min, and absorbance was measured spectrophotometrically at 510 nm to quantify the neutral lipids (24).

For the quantification of phospholipids, only viable cells were collected and resuspended in a 20:80 (v/v) methanol:acetonitrile mixture (0.3 ml). Aliquots of the cells´ extracts were saved for protein quantification. The analysis was performed on an Agilent 1260 HPLC system (Agilent) equipped with a Photodiode array and an UV-VIS multivariate wavelength detector. The column used in these analyses was a C18 4.5 μm particle, 2.1 × 50mm (Poroshell, Agilent). The mobile phases consisted of deionised water, 18 MΩcm (A) and acetonitrile (B), both containing 0.1% (v/v) formic acid. Aliquots (5 μl) of the cell extracts were injected in each cycle and the analysis was performed with the following gradient (0.8 ml/min): 20% B for 2 min to 100% B in 16 min, held at 100% for 7 min. Three independent samples were analysed (three replicates). Identification of the different compounds was attained by matching retention times and co-injection of the standards.

### 2.9 Patient recruitment and gene expression analysis in PBMCs

The clinical trial was approved by the Ethics Committee for Clinical Investigation of La Paz University Hospital (Ref. HULP 5617), and it was carried out in accordance with The Code of Ethics of The World Medical Association (Declaration of Helsinki). Written informed consent was obtained by all subjects prior to starting the trial. The ClinicalTrials.gov Identifier: NCT05080920.

Eight NSCLC patients were recruited at the Medical Oncology Service of Infanta Sofia University Hospital (San Sebastian de los Reyes, Madrid, Spain) from November 27, 2020, to March 24, 2021. Clinical and pathological data were collected from medical reports (**Supplementary Table 2**). The set-up of the pilot clinical trial was a sixteen week, double-blind, randomised, and parallel pilot study with two study arms: SFRE formulated with alkylglycerols capsules (CR) (PCT/ES2017/070263), and control capsules (CC). Composition of CR and CC capsules were previously described (25).

The main objective of the study was to evaluate the effects of the intervention on the expression of a selected panel of genes related to lipid metabolism, inflammation, oxidative stress, immune system, and oncogenic pathways in PBMCs. A Taq-Man Low Density Array (Applied Biosystems, Madrid, Spain) was specifically designed for this experiment, including 46 selected genes (**Supplementary Table 3**) linked to immune system, inflammation, oxidative stress, lipid metabolism, and lipid related oncogenic genes.

Patients were instructed to fast overnight before each blood collection. Blood samples were collected in heparinized tubes (BD Vacutainer, Franklin Lakes, NJ, USA) at each visit between 08:00 and 10:00 to minimize circadian variations, and there were processed within 2 h of collection for the isolation of the PBMCs. Isolation was carried out under sterile conditions to avoid monocytes activation. Briefly, whole blood was diluted (1:1) with phosphate buffer solution (PBS) and centrifuged by density gradient with Histopaque-1077 (Sigma–Aldrich, Madrid, Spain), according to the manufacturer’s instructions. After collection, PBMCs were washed twice with PBS.

It has been compared the evolution of gene expression in PBMCs from the initial visit (V1) to visit 4 (nine weeks of intervention) and to visit 6 (sixteen weeks of intervention). Intermediate visits V2, V3 and V5 were control visits.

Gene-expression assays were performed in a HT–7900 Fast Real-Time PCR. *B2M* and *18S* genes were used as endogenous controls. RT-StatMiner software (Integromics^®^ Inc., Madison, WI, USA) was used to detect and determine the quality control and differential expression analyses. The Expression Suite Software (Life Technologies, Madrid, Spain) program was used to obtain the Ct data. The ΔCt (Ct gene-Ct endogenous gene) was calculated, and then the relative expression (RQ) between visits was calculated (V4 *vs* V1, and V6 *vs* V1) following the 2- ^-ΔΔCt^ method (23).

### 2.10 Statistical analysis

Gene expression in NSCLC cells were analysed by non-parametric ANOVA with Bonferroni *post hoc* tests. Data were represented as mean ± S.E.M of at least three independent experiments. Statistical differences were defined as *p* < 0.05 (*); *p* < 0.01 (**); *p* < 0.005 (***); p < 0.001 (****). Statistical analysis was performed with Graph Pad Prism 8 software (Version 8.0.0, GraphPad Software, San Diego, CA, USA).

Gene expression in PBMCs from NSCLC patients was quantified with the ΔCt method as previously described (26). To ease interpretation of the results, data was sign-reversed so that lower ΔCt values corresponded to lower gene expression levels. A mixed linear model was used to test the differential evolution of gene expression through time (baseline V1, V4-nine weeks and V6-sixteen weeks) for the SFRE-treated patients *vs* the control-treated ones, adjusted for sex and age. Time and treatment were modelled as fixed interacting effects, and patient as random effect. P. values were corrected by the Bonferroni method to deal with multiple test type-I error inflation due to the multiple genes tested. Statistical significance was defined as P-value < 0.05 with bilateral tests, and 95% confidence intervals for estimated parameters were calculated.

The statistical analyses were performed using the R statistical software version 3.6.1 (www.r-project.org).

## 3 Results

### 3.1 SFRE inhibits cell proliferation of NSCLC cell lines

Previously, SFRE has been demonstrated to inhibit lipid metabolism in colorectal and breast cancer cell lines (17, 19, 27). Due to the relevance of the altered lipid metabolism in lung cancer (14, 28, 29), herein, we aimed to investigate the potential of SFRE on the inhibition of lipid metabolism in NSCLC.

Using the available data from the Cancer Cell Line Encyclopedia https://portals.broadinstitute.org/ccle/about) and COSMIC (https://cancer.sanger.ac.uk/cosmic) databases, we investigated the expression levels of *SREBF1, SCD* and *FASN* to select two NSCLC cell lines with intermediate gene expression levels for the three genes. As epidermal growth factor receptor (EGFR) is a key tumour driver and one of the main targets in the successful treatment of NSCLC (3, 4), and as T790M and L858R mutations have been related to the acquisition of resistance to EGFR tyrosine kinase inhibitors in the clinical setting (30), we decided to use H1299, which is *EGFR* wild type, and H1975, which presents the indicated mutations in EGFR, for the *in vitro* studies.

First, we analysed the effect of SFRE on the cell proliferation of the two NSCLC cell lines -H1299 and H1975- by mean of the MTT assay.

**Figure 1:**
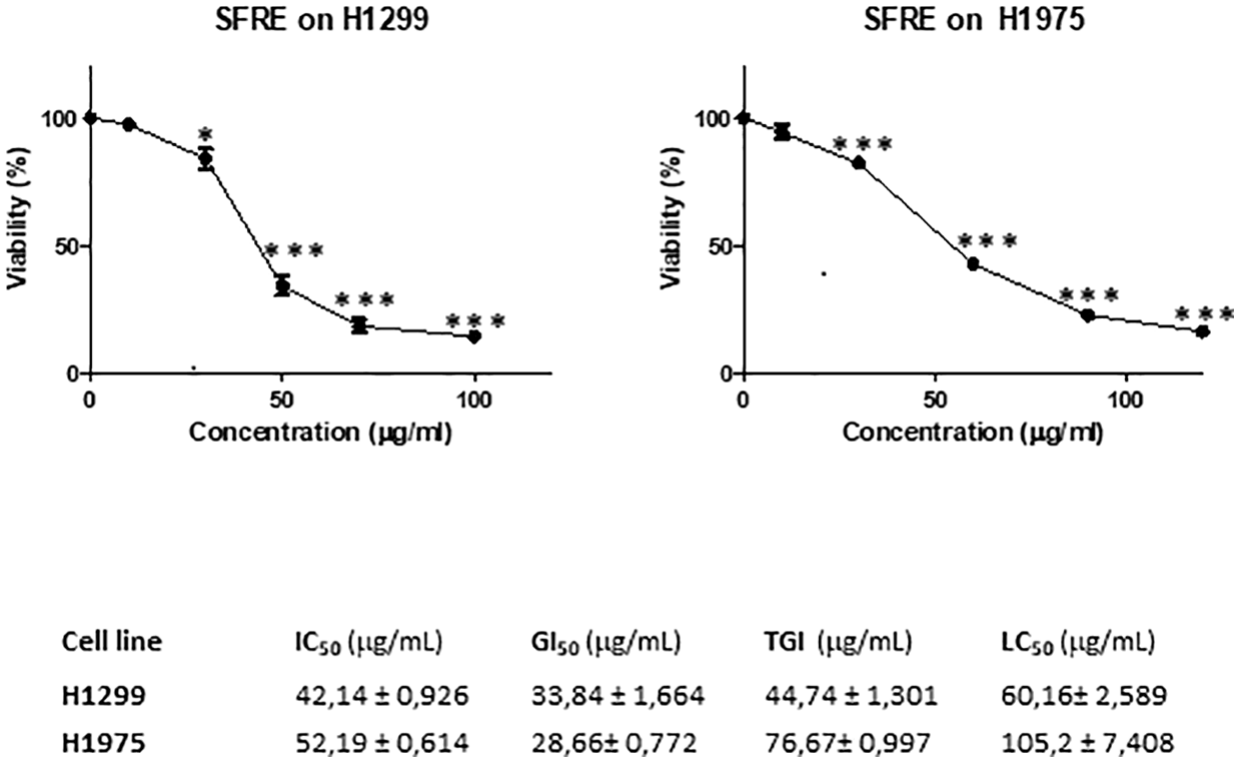
SFRE inhibits cell proliferation of NSCLC cell lines -H1299 and H1975. Dose–response curves of the cell proliferation assay after 48 h of treatment with increasing concentrations of SFRE. Data represent mean ± SEM of three independent experiments, each performed in triplicates. Values corresponding to IC_50_, GI_50_, TGI and LC_50_ after 48 h of SFRE treatment are indicated. Results are shown as the mean ± SEM of three independent experiments, with 4 replicates/experiment. Asterisks * and *** indicate *p* values < 0.05 and 0.005, respectively.

As shown in **Figure 1**, SFRE diminished the cell proliferation of both cell lines in a dose dependent manner. Concentrations corresponding to 50% of cell proliferation inhibition (IC_50_), 50% growth inhibition (GI_50_), total growth inhibition (TGI), and 50% cell death (LC_50)_ are also shown.

H1299 was more sensitive to SFRE treatment as shown by the lower IC_50_ values obtained compared to H1975 which may be related to the different functional status of EGFR.

### 3.2 SFRE inhibits cell bioenergetics of NSCLC cell lines

It is well known that cancer cells adapt their metabolism to support cell proliferation, progression and/or resistance to chemotherapy. As SFRE inhibited the cell proliferation of NSCLC cells, we wanted to investigate if SFRE was able to diminish the main bioenergetic pathways, this is, mitochondrial oxidative phosphorylation and aerobic glycolysis of NSCLC cells. H1299 and H1975 NSCLC cells were pre-treated for 48 h with three different doses of SFRE, based on the previously determined values IC_50_ (**Figure 1**). Then, we quantified, by flux analysis of the extracellular media, the Extracellular Acidification Rate (ECAR) and the Oxygen Consumption Rate (OCR), as main readouts of aerobic glycolysis and mitochondrial oxidative phosphorylation, respectively.

### 3.3 SFRE diminishes aerobic glycolysis of NSCLC cells: GlycoStress assay

**Figure 2:**
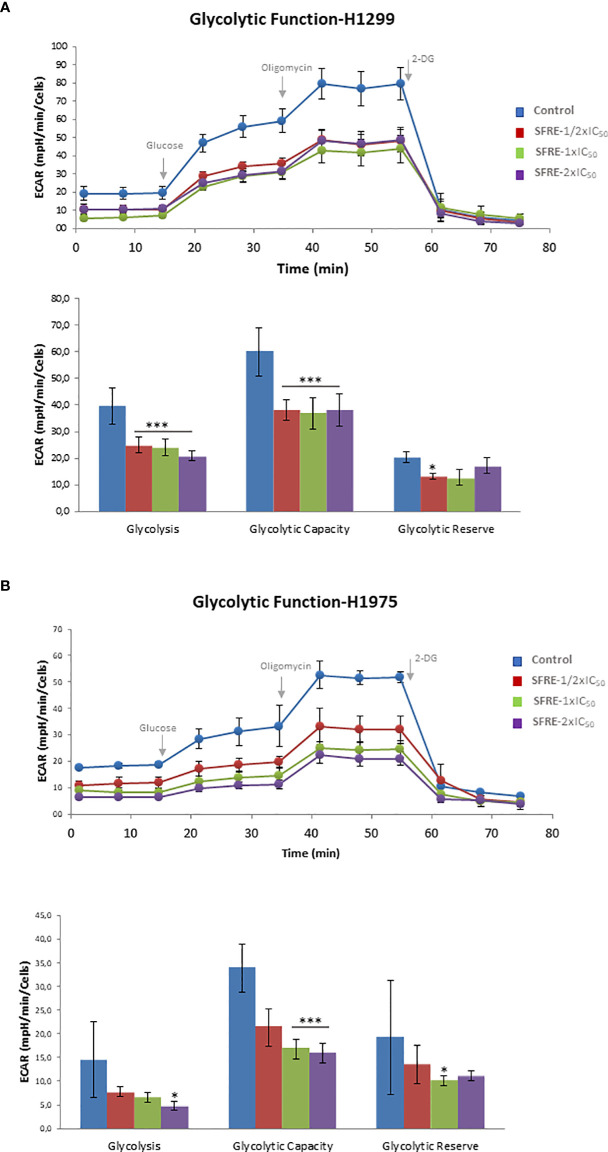
SFRE diminishes aerobic glycolysis. Aerobic glycolysis analysis by the flux analysis of the extracellular acidification rate (ECAR) of H1299 cells **(A)** and H1975 **(B)**, previously pre-treated with SFRE at three doses (1/2xIC_50_, 1xIC_50_ and 2xIC_50_) for 48 h The basal ECAR, glycolysis and maximal ECAR of 10,000 cells per condition are compared. Data represent mean ± SEM of three independent experiments, each performed four to six replicates. Asterisks *, *** indicate p-values < 0.05, and 0.005, respectively.

Highly proliferative cancer cells frequently upregulate aerobic glycolysis independently of oxygen availability (*Warburg effect*). For this reason, we first analysed the aerobic glycolysis performance of NSCLC cells after 48 h of treatment with SFRE, by the monitorization of the Extracellular Acidification Rate (ECAR), which is an indirect readout of the L-lactate production by the cells. We previously pre-treated H1299 and H1975 cells for 48 h with three different doses of SFRE corresponding to 1/2xIC_50_, 1xIC_50_ and 2xIC_50_. Non-treated cells were kept as controls. Importantly, after SFRE treatment, the very same number of cells (10,000 cells/well) were plated in a XFe-plate in the absence of SFRE extract. Cells were kept in the incubator for 6 h to allow the cells to attach to the plates. Then, the medium of the cells was changed to the non-buffered XFe Base media (pH 7.4), supplemented with 2 mM glutamine, in the absence of glucose, for 30 min at 37°C without CO_2_. We first monitored the basal ECAR as an indirect readout of the basal L-lactate production by aerobic glycolysis. Interestingly, basal ECAR of NSCLC cells (three measurements before oligomycin injection) was diminished in SFRE treated cell compared to that of non-treated cells (**Figure 2**). Next, we injected glucose to the medium to monitor cells´ ability to upregulate aerobic glycolysis when glucose is available. After the injection of glucose, although NSCLC cells were able to respond to glucose, they showed diminished levels of ECAR compared to that of non-treated control cells. When oligomycin was added to block ATP production from mitochondria, maximal ECAR of NSCLC cells pre-treated with SFRE was clearly reduced compared to non-treated cells. Finally, 2-deoxyglucose, a glucokinase competitive inhibitor, was injected to quantify the ECAR specifically associated to aerobic glycolysis. Quantification of main parameters of glycolytic activity indicated that glycolysis, glycolytic capacity and glycolytic reserve were inhibited in the pre-treated H1299 and H1975 NSCLC cells, in line with the reduction of the gene expression levels of *GLUT4* in both cell lines and *LDHA* in H1299 (**Supplementary Figure 1**).

These results indicate that SFRE inhibits aerobic glycolysis of NSCLC cells.

### 3.4 SFRE diminishes mitochondrial oxidative phosphorylation: MitoStress assay

Due to the observed effects of SFRE in the inhibition of the aerobic glycolysis, we next investigated if SFRE had any effect on the mitochondrial oxidative phosphorylation. First, we monitored OCR in response to well defined modulators of mitochondrial function by mean of the MitoStress assay.

Before running the MitoStress assay, the very same number of non-treated cells and previously pre-treated cells were plated in a XFe Seahorse plate in complete media (RPMI, 10% FBS), and kept for 6 h to allow the cells to attach in the absence of any treatment, to compare the cell bioenergetic profile only of viable cells. Then, the media of the cells was changed to the non-buffered XFe Base media, pH 7.4, supplemented with 10 mM glucose, 2 mM glutamine, and 1 mM pyruvate, and cells were incubated for 30 min at 37°C without CO_2_.

**Figure 3:**
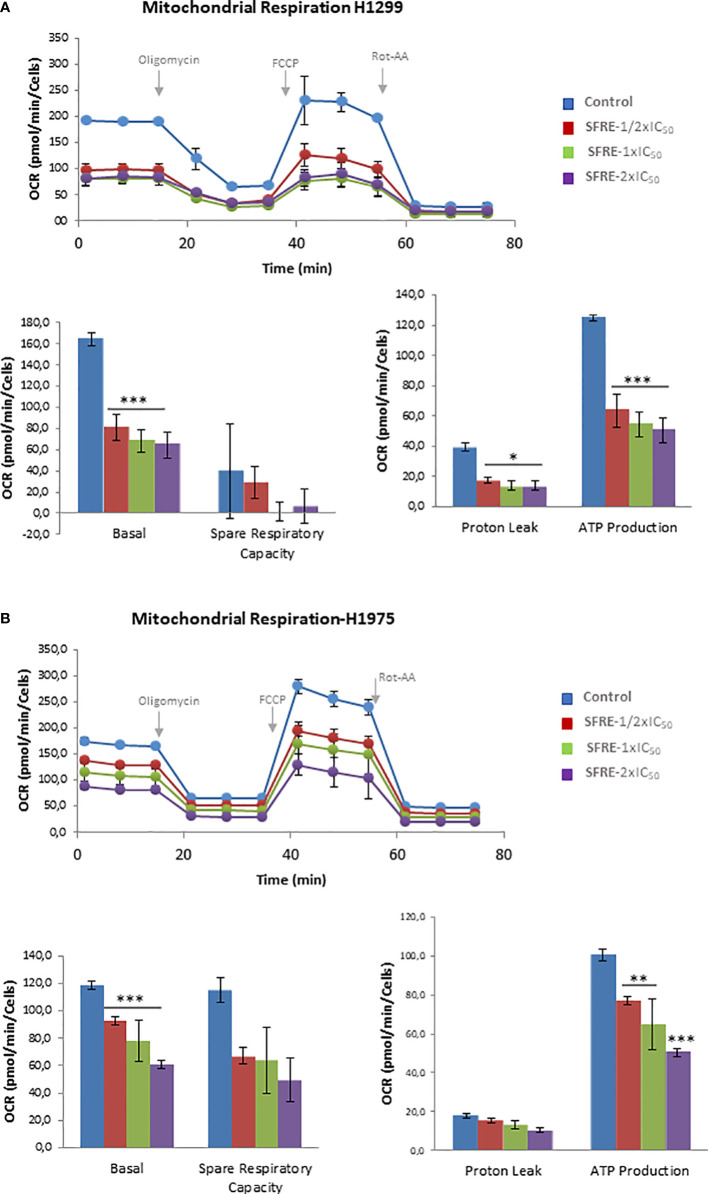
SFRE diminishes mitochondrial oxidative phosphorylation. Mitochondrial respiration analysis by flux analysis of the oxygen consumption rate (OCR) of H1299 cells **(A)** and H1975 **(B)** previously pre-treated with SFRE (1/2xIC_50_, 1xIC_50_ and 2xIC_50_) for 48 h The basal respiration rate, spare respiratory capacity, ATP production, and proton leak of 10,000 cells per condition are compared. Data represent mean ± SEM of three independent experiments, each performed with four to six replicates. Asterisks *, **, *** indicate *p* values < 0.05, 0.01 and 0.005, respectively.

As it is shown in **Figure 3A** H1299 NSCLC cells pre-treated with SFRE displayed reduced basal respiration rates (BRR) compared to control non-treated cells (measurements 1 to 3) at all the doses tested. After the oligomycin injection, SFRE pre-treated cells showed reduced levels of ATP compared to control non-treated cells (measurements 4 to 6). The maximal respiration rate (MRR) (measurements 7 to 9), after the injection of FCCP, was also affected in SFRE pre-treated cells. Finally, rotenone and antimycin A, inhibitors of complexes I and III of the electron transport chain (ETC) respectively, were injected to shut down the OCR due to mitochondrial oxidative phosphorylation (measurements 10 to 12). These results indicate that SFRE compromises mitochondrial respiration. Similar results were obtained in H1975 cells (**Figure 3B**).

The effects of SFRE on the inhibition of the main bioenergetic pathways in NSCLC cells seemed to be on the bases of the observed effects on the inhibition of cell proliferation, as indicated by the quantification of the intracellular ATP content (**Supplementary Figure 2A**), and the upregulation of gene expression of apoptotic markers (*Caspase-9* and *DDIT3*) (**Supplementary Figure 2B**).

### 3.5 SFRE inhibits lipid metabolism related genes in NSCLC cell lines

Due to the observed effects of SFRE on cellular metabolism and, taking into consideration that SFRE diminished lipid metabolism in colorectal cancer (CRC), we wanted to evaluate if SFRE may affect lipid metabolism in NSCLC (25, 31).

With this objective we designed a panel of metabolic genes -including *de novo* lipogenesis and cholesterogenesis (*SREBF1, FASN, SCD and HMGCR*), fatty acid metabolism (*ACSL1, ACSL3, ACSL4, AGPAT1)*, exogenous uptake of lipids (*LDLR)* regulation of intracellular cholesterol levels (*ABCA1, ApoA1*), oncogenic pathways (*CHKA, EGFR*), inflammation and oxidative stress (*JAK1, NEF2L2*)-.

**Figure 4:**
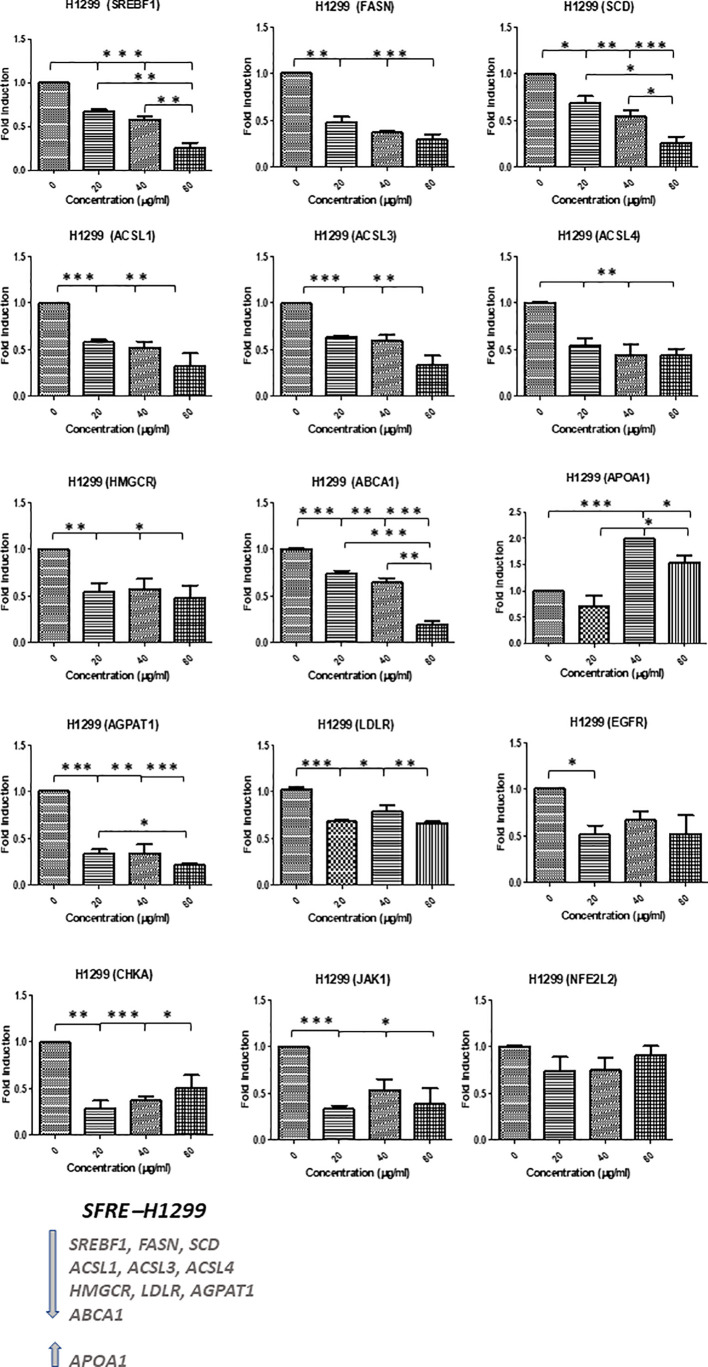
Effects of SFRE on lipid metabolism related genes after 48 h of treatment H1299 NSCLC cells. SFRE diminishes the expression of *SREBF1, FASN, SCD1, HMGCR* (*de novo* lipogenesis and cholesterogenesis); *ACSL1, ACSL3* and *ACSL4* (fatty acid activation); *ABCA1* (efflux of cholesterol and tumour microenvironment remodelling); *CHKA* and *AGPAT1* (biosynthesis of phospholipids and plasmatic membrane phospholipid remodelling) and oncogenic *EGFR*. On the contrary, SFRE upregulated the expression of *APOA1*. Results are shown in relation to non-treated cells and normalized to the endogenous control *B2M*. Results are expressed as the mean ± SEM of three independent experiments, each performed in triplicates. Asterisks *, **, *** indicate *p* values < 0.05, 0.01 and 0.005, respectively.

SFRE diminished the expression of the master regulator *SREBF1* implicated in the *de novo* lipogenesis and its downstream genes, *FASN* and *SCD*, as well as *HMGCR* implicated in the *de novo* cholesterogenesis, in H1299 cells (**Figure 4**). In addition, the expression of *LDLR*, implicated in the uptake of extracellular lipids, together with *ABCA1*, implicated in the reverse transport of cholesterol, were also downregulated. Targets related to the synthesis of phospholipids (*CHKA*), phospholipid remodelling at membranes (*AGPAT1*), oncogenic pathways (*EGFR)* and inflammation (*JAK1*) were also downregulated. Interestingly, SFRE upregulated the expression of *APOA1* which has been proposed to be a tumour suppressor in several cancers (32).

**Figure 5:**
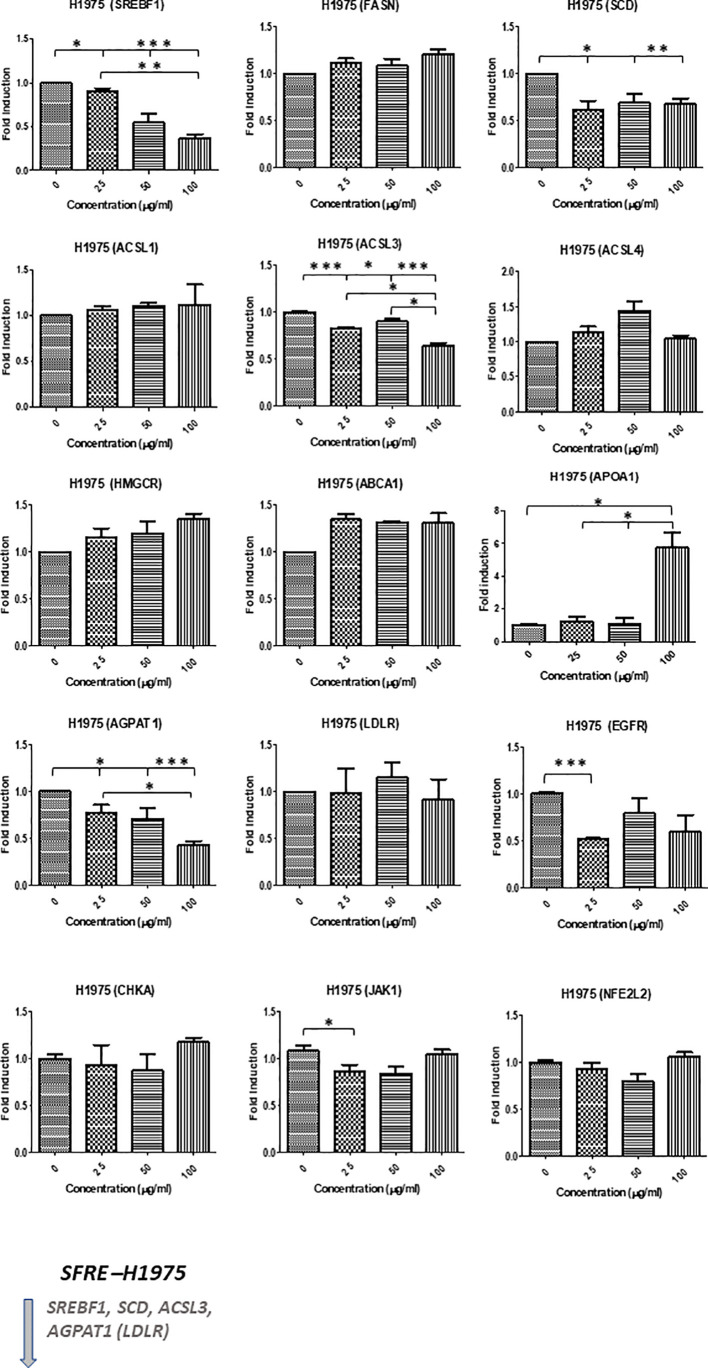
Effects of SFRE on lipid metabolism related genes after 48 h of treatment H1975 NSCLC cells. SFRE diminishes the expression of *SREBF1, FASN, SCD1, HMGCR* (*de novo* lipogenesis and cholesterogenesis); *ACSL1, ACSL3* and *ACSL4* (fatty acid activation); *ABCA1* (efflux of cholesterol and tumour microenvironment remodelling); *CHKA* and *AGPAT1* (biosynthesis of phospholipids and plasmatic membrane phospholipid remodelling) and oncogenic *EGFR*. On the contrary, SFRE upregulated the expression of *APOA1*. Results are shown in relation to non-treated cells and normalized to the endogenous control *B2M*. Results are expressed as the mean ± SEM of three independent experiments, each performed in triplicates. Asterisks *, **, *** indicate *p* values < 0.05, 0.01 and 0.005, respectively.

Similar results were obtained in H1975, although this cell line, EGFR mutated, seemed to be less sensitive to SFRE treatment (**Figure 5**).

### 3.6 Neutral lipids content and quantification of membrane phospholipids

Due to the observed effects of SFRE in the expression of lipid metabolism genes, we wanted to evaluate the biological relevance of these effects by quantifying the intracellular neutral lipid content and main membrane phospholipids. We previously pre-treated H1299 and H1975 cells for 48 h with three different doses of SFRE corresponding to 1/2xIC_50_, 1xIC_50_ and 2xIC_50_. Non-treated cells were kept as controls. Then, cells were kept in the incubator for 6 h to allow the cells to attach to the plates.

**Figure 6:**
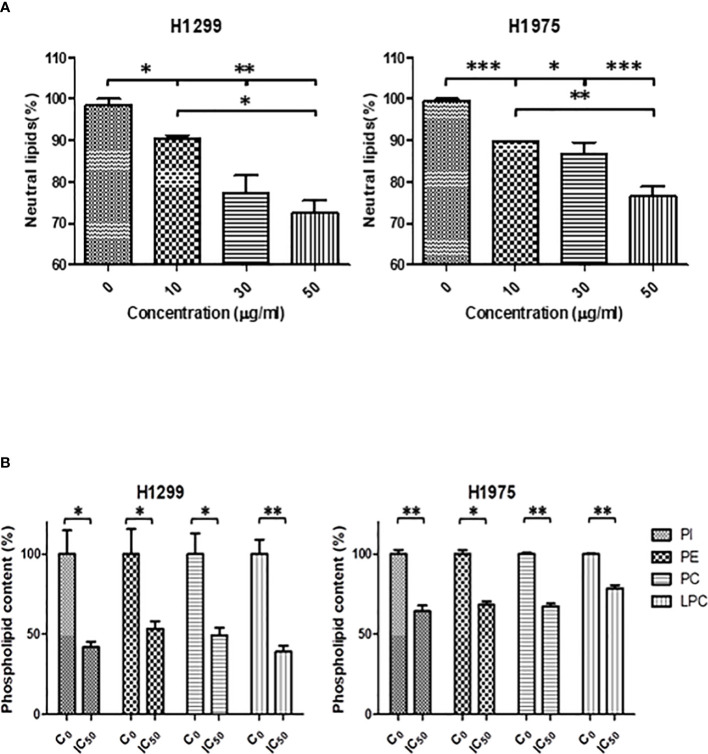
**(A)** SFRE reduced the intracellular neutral lipids content of H1299 and H1975 cells after 48 h of treatment at the indicated doses. Relative content of neutral lipids in cells pre-treated with SFRE after 48 h at different doses, compared with untreated control cells, normalized by the number of viable cells. Results are shown mean ± SEM of three independent experiments (three replicates). **(B)** SFRE reduced the total content of main phospholipids. Relative content of phospholipids in cells pre-treated with SFRE after 48 h at the fixed dose of IC_50_ for each cell line, compared with untreated control cells, normalized by protein content. Results are shown mean ± SEM of three independent experiments (three replicates). Asterisks *, **, *** indicate *p* values < 0.05, 0.01 and 0.005, respectively.

As shown in **Figure 6A** the total neutral lipid content was diminished in a dose dependent manner after SFRE treatment in H1299 and H1975 cell lines. In line with these results, SREBP1 levels, quantified by FACs analysis, were also found diminished (**Supplementary Figure 3**).

In addition, the quantification of main species of phospholipids indicated that SFRE reduced the levels of phosphatidyl-inositol (PI), phosphatidyl-ethanolamine (PE), phosphatidyl-choline (PC) and the lyso-forms (LPC) in both NSCLC cell lines (**Figure 6B** ).

### 3.7 SFRE augments NSCLC sensitivity to chemotherapeutic drugs and immune checkpoint inhibitors used in the clinic

In almost all of stage I and stage II NSCLC patients, surgery remains the standard treatment in the clinic. Stage III cases involves a multidisciplinary therapy with a combination of chemo, radiation and, in PD-1 >1%, immunotherapy; Stage IV patients could be treated with immunotherapy or a chemo-immunotherapy according to PD-L1 expression of the tumor (33). Since dual therapy with platinum agents and other drugs, including taxanes or pemetrexed has been widely used as standard treatment for advanced NSCLC without specific mutations, attention has focused on combining these regimens with immunotherapy (34, 35).

An improvement in the understanding of the immunology of cancer and the tumour microenvironment has allowed the application of immunotherapy (36).

#### 3.7.1 SFRE inhibits NSCLC cell proliferation synergistically in combination with cisplatin

**Figure 7:**
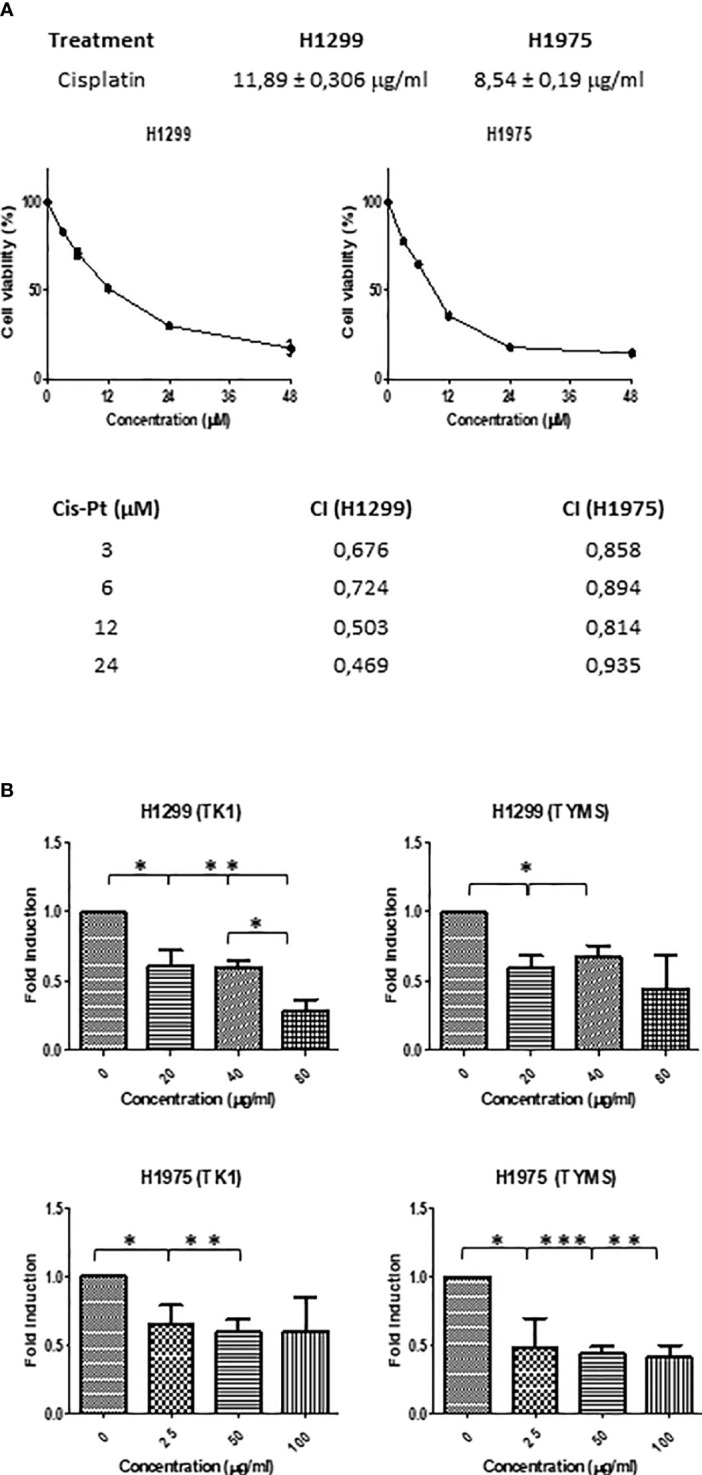
**(A)** Dose–response curves of the cell proliferation after 48 h of treatment with cisplatin in NSCLC H1299 and H1975. IC_50_ values after cisplatin treatment in the two different NSCLC cell lines (H1299 and H1975) after 48h of treatment are indicated (upper panel). SFRE synergised with cisplatin in the inhibition of NSCLC cell proliferation. NSCLC cells were pre-treated with the extract for 3h at a fixed dose of 1/2 IC_50_ for H1299 and H1975. Combinatory index (CI) according to Chou–Talalay method (lower panel). **(B)** SFRE diminished the expression of *TK1* and *TYMS*, in a dose dependent manner. Results are shown in relation to non-treated cells and normalized to the endogenous control *B2M*. Results are expressed as the mean ± SEM of three independent experiments, each performed in triplicates. Asterisks *, **, *** indicate p-values < 0.05, 0.01 and 0.005, respectively.

As indicated, cisplatin is one of the most common standard first-line treatments, excluding targeted therapies. For this reason, we aimed to evaluate if SFRE could act synergistically with cisplatin in the inhibition of the cell proliferation of NSCLC cells. First, we calculated the IC_50_ values of cisplatin on NSCLC cell lines (**Figure 7A**, upper panel).

Importantly, the combination index (CI) after treatment NSCLC cell lines with SFRE for three hours at a fixed dose of 1/2 IC_50_ for H1299 and H1975, indicated a positive synergism between cisplatin and SFRE (**Figure 7A**).

In a previous work, we have demonstrated that SFRE inhibited thymidine kinase 1 (*TK1*) and thymidylate synthase (*TYMS*) synergising with 5- fluorouracil (5-FU) in the inhibition of colorectal cancer cell lines proliferation (19). The analysis of the expression of these genes in NSCLC cells after the treatment with SFRE indicated the downregulation of *TK1* and *TYMS* (**Figure 7B**) which may explain, at least partially, the observed synergism in the inhibition of cell proliferation between SFRE and cisplatin.

#### 3.7.2 SFRE inhibits NSCLC cells proliferation synergistically in combination with pemetrexed

**Figure 8:**
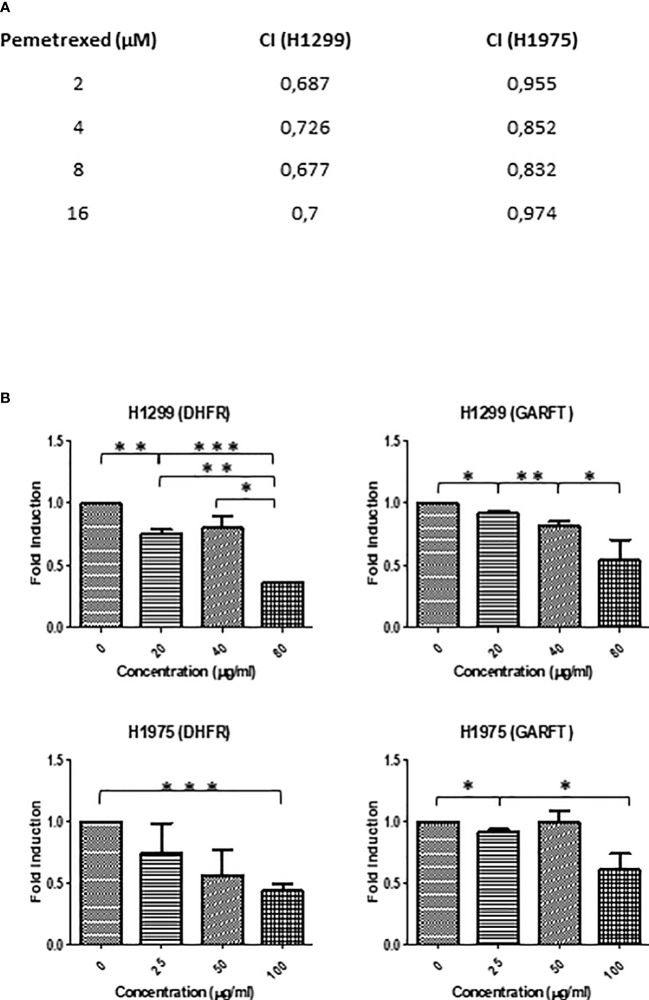
**(A)** SFRE synergised with pemetrexed in the inhibition of NSCLC cell proliferation. NSCLC cells were pre-treated with the extract for 3h at a fixed dose of 1/2 IC_50_ for H1299 and H1975. Combinatory index (CI) according to Chou–Talalay method. **(B)** SFRE diminished the expression of *DHFR* and *GARFT*, in a dose dependent manner. Results are shown in relation to non-treated cells and normalized to the endogenous control *B2M*. Results are expressed as the mean ± SEM of three independent experiments, each performed in triplicates. Asterisks *, **, *** indicate p-values < 0.05, 0.01 and 0.005, respectively.

During the last decade, the place of pemetrexed for the treatment of non-squamous NSCLC became established. Pemetrexed belongs to the ‘folate antimetabolites’ class of chemotherapy agents, and it inhibits cell replication and tumor growth by inhibiting the activity of three enzymes involved in purine and pyrimidine synthesis: thymidylate synthase (*TYMS*), dihydrofolate reductase (*DHFR*) and glycinamide ribonucleotide formyl-transferase (*GARFT*) (37). For this reason, we aimed to evaluate if SFRE could act synergistically with pemetrexed in the inhibition of the cell proliferation of NSCLC cells. Taking into consideration the IC_50_ values described for pemetrexed in the inhibition of cell proliferation of H1299 [4.72 ± 1.9 µM (38) and 2.43 µM (39)] and the described IC_50_ values for H1975 (3.372 ± 0.082 µM (40) and 3.37 ± 0.14 µM (41), we pre-treated NSCLC cell lines with SFRE for three hours at a fixed dose of 1/2 IC_50_ for H1299 and H1975, and then a range of concentrations of pemetrexed from 2 to 8 ug/ml were used concomitantly with SFRE. Importantly, the combination index (CI) indicated a positive synergism between pemetrexed and SFRE (**Figure 8A**). The analysis of the expression of dihydrofolate reductase (*DHFR*) and glycinamide ribonucleotide formyl-transferase (*GARFT*) genes in NSCLC cells after the treatment with SFRE, indicated the downregulation of both genes (**Figure 8B**), which may explain, at least partially, the observed synergism between SFRE and pemetrexed.

#### 3.7.3 SFRE inhibits NSCLC cells proliferation synergistically in combination with pembrolizumab

Immunotherapy has made the breakthrough in cancer therapeutics, as it aims to modulate immune regulatory mechanisms to enhance the immune response against cancer cells. The introduction of the monoclonal antibodies anti-PD-1 and anti-PD-L1 has significantly changed the landscape of the treatments for advanced non-small-cell lung cancer (NSCLC). Clinical studies evaluating the response to immunotherapy in patients have demonstrated superior survival indexes as well as reduced toxicity profiles in comparison to standard chemotherapy regimens. Of note, anti-PD-1 therapy (pembrolizumab) has recently replaced chemotherapy in the first line treatment for NSCLC with high PD-L1 expression, and the addition of pembrolizumab to platinum chemotherapy resulted in a significant improvement in overall survival (OS) in patients with non-squamous NSCLC, regardless of PD-L1 expression. Although PD-1 is mainly expressed on the activated T cells, B cells, and monocytes, recent studies have shown that PD-1 is expressed in a subpopulation of various cancer cells, including melanoma, hepatocellular carcinoma (HCC) and NSCLC (42). In the absence of adaptive immune system, tumor cell-intrinsic PD-1/PD-L1 mediates the resistance to anti-PD-1/PD-L1 antibodies by activating AKT and ERK1/2. These findings provide an additional explanation for resistance to cancer immunotherapy. As pembrolizumab interacts with PD-1, we hypothesised that blocking the intracellular signalling could also affect the cell proliferation of NSCLC cells, as described by mean of the inhibition of the canonical signalling pathways, i.e. the AKT and ERK1/2 pathways (43).

For this reason, we evaluated if SFRE could act synergistically with pembrolizumab in the inhibition of the cell proliferation of NSCLC cells.

**Figure 9:**
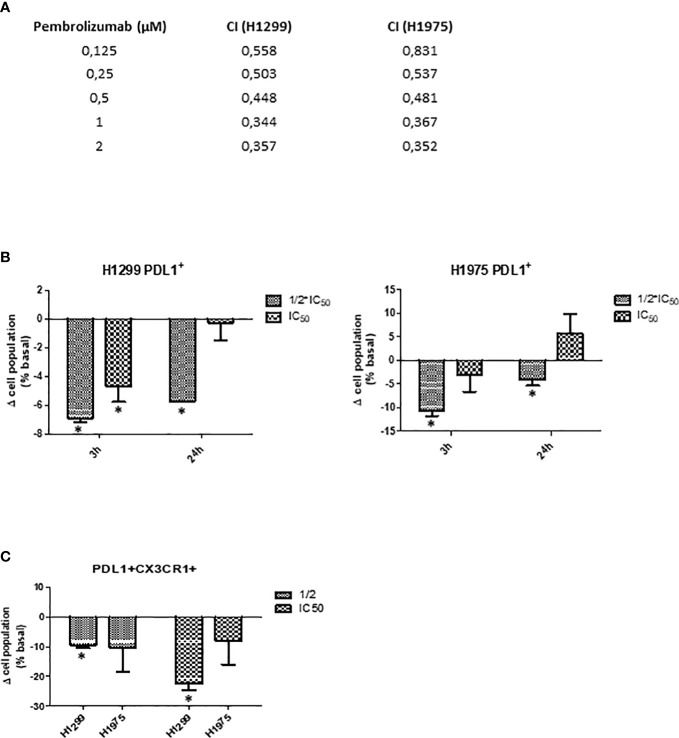
SFRE synergised with pembrolizumab in the inhibition of NSCLC cell proliferation. H1299 and H1975 NSCLC cells were treated concomitantly with SFRE at a fixed dose of 1/2xIC_50_ and with pembrolizumab at the indicated doses. **(A)** The combinatory index (CI) according to Chou–Talay method indicated a positive synergism between SFRE and pembrolizumab in the inhibition of NSCLC proliferation. **(B)** FACs analysis of the expression of PD-L1 after 3h and 24h SFRE treatment of NSCLC cells at two different doses (1/2xIC_50_, and 1xIC_50_. **(C) **FACS analysis of the double expression of PD-L1 and CXCR1 after 3h and 24h SFRE treatment of NSCLC cells at two different doses (1/2 IC50, and 1xIC50. Data are expressed as fold induction respect to the expression levels of non-treated cells. Results are expressed as the mean ± SEM of three independent experiments, each performed in triplicates. Asterisks * indicates p-values < 0.05.

Considering a range of concentrations for pembrolizumab from 0.12 to 2μM, based on the described IC_50_ values of pembrolizumab in the inhibition of cell proliferation of the NSCLC A549 cell line (EGFR wt) (IC_50 =_ 0.4 µM) (44), we treated NSCLC cell lines concomitantly with SFRE at a fixed dose of 1/2 IC_50_. As shown in **Figure 9A**, the combination index (CI) indicated a positive synergism between pemetrexed and SFRE in the inhibition of NSCLC cell proliferation (**Figure 9A**).

To investigate the underlying mechanisms for the observed synergism and taking into consideration that the interaction of pembrolizumab with PD-1 occurs between 30 min and 3h of treatment, we analysed by flow cytometry the expression of PD-L1 after SFRE treatment for 3h and 24h. As shown in **Figure 9B**, SFRE diminished the expression of PD-L1, being this effect higher at the lowest doses (1/2 xIC_50_) and at the shorter time of treatment (3h). As PD-L1 expression in tumours has been described to augment the expression of other immunosuppressive biomarkers such as CX3CR1 at the local tumour microenvironment (45), we also quantified the double expression of PD-L1 and CX3CR1 after SFRE treatment. SFRE diminished the expression of PD-L1 and CX3CR1 as shown in **Figure 9C**, which indicates the potential of SFRE ameliorating the expression of biomarkers that promote immune evasion of the lung cancer cells.

#### 3.7.4 SFRE modulates the expression of lipid metabolism related targets in NSCLC patients

In a previous work, we conducted a nutritional trial in healthy volunteers with an SFRE extract formulated with alkylglycerols (AKG) to increase the bioavailability of the bioactive compounds from SFRE after gastrointestinal digestion (PCT/ES2017/070263). Importantly, it was demonstrated not only the tolerability and safety of the intervention, but also the potential therapeutic action of SFRE by mean of the activation of innate immunity and the modulation of the expression of genes related to immune system, inflammation, oxidative stress, and cancer (25).

Due to the observed effects of SFRE in NSCLC cells related to the inhibition of lipid metabolism together with its synergism with chemotherapeutic drugs used in the treatment of NSCLC patients, herein, we wanted to investigate the clinical relevance of SFRE as a putative co-adjuvant in the treatment of NSCLC patients.

In collaboration with the Medical Oncology Service of Infanta Sofia University Hospital, PBMCs were obtained from eight NSCLC patients.

To evaluate if genes related to inflammation, oxidative stress, and immune system as readouts of changes at the systemic level may be affected as readouts of changes at the systemic level, may be affected or not, we compared the evolution of gene expression from V1 to visit 4 (nine weeks of intervention) and to visit 6 (sixteen weeks of intervention), in the two groups of patients: SFRE-intervention group (CR) *vs* control (CC).

**Supplementary Table 3** shows the panel of genes analysed and the metabolic pathways where they are implicated).

Thus, it was evaluated the differential time evolution of gene expression in PBMCs from the initial visit (V1) to visit 4 (nine weeks of intervention) and to visit 6 (sixteen weeks of intervention), in the two groups of patients: SFRE-treated (CR) vs control (CC).

**Figure 10:**
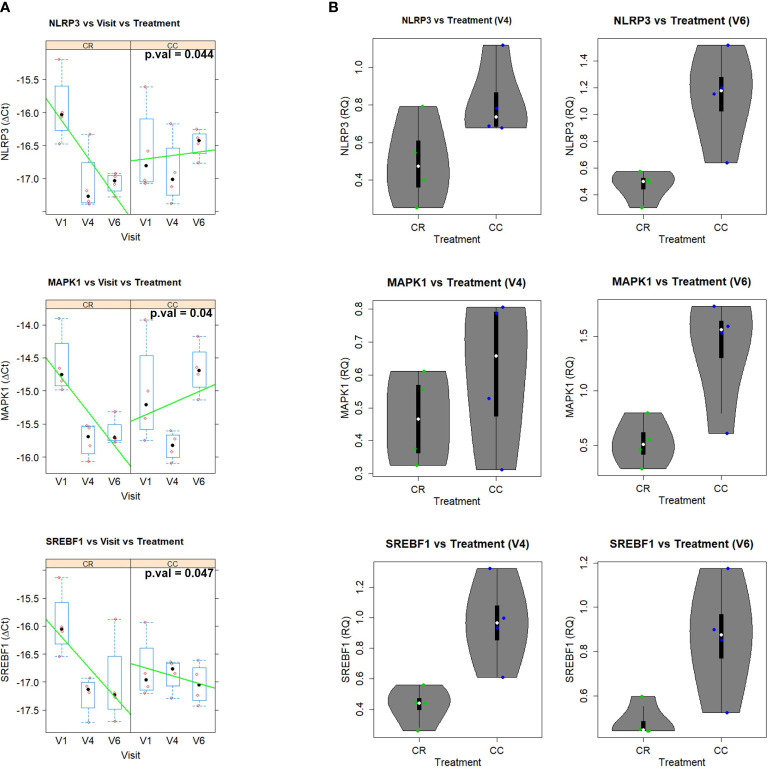
**(A)** Gene expression (DCt) patterns across time (V1 (baseline), V4 (nine weeks of intervention) and V6 (sixteen weeks of intervention), for the significant genes in the two groups: CR (SFRE treatment) and CC (control). Boxplots are displayed for each visit and group, together with the actual data points. Line trends are added in green to ease visualization of the trends. The corresponding p.values (after multiple test correction) are: 0.044, 0.04, and 0.047, for NLRP3, MAPK1, and SREBF1, respectively. **(B)** Violin plots showing the fold change (RQ = 2^-DDCt) of V4 and V6 in comparison to V1 of the statistically significant genes. Boxplots are also shown together with the actual data points.

The analysis of gene expression (ΔCt) through the different visits and treatments indicated a statistically significant visit x treatment interaction after multiple test correction for *MAPK (p=0.04)*, *NLRP3 (p=0.044)*, and *SREBF1 (p=0.047)* (**Figure 10A** and **Supplementary Table 4**).

The two treatments displayed very different time trends, with the CR group showing a strong decrease of gene expression with time in the three genes while the CC control showing an increase of *NLRP3* or slight decrease of *SREBF1* (**Figure 10B**). More specifically, the analysis in terms of the relative gene expression (RQ = 2^-ΔΔCt^) of visits 4 and V6 relative to visit 1 indicated that *NLRP3, MAPK, SREBF1* displayed fold inductions below 1 in the CR group, corresponding to inhibition of gene expression, while in the case of the control CC group the fold induction was above 1, indicating an exacerbated gene expression **Figure 10B**).

Data suggest that CR group displayed a better control of lipid metabolism related inflammatory pathways at the systemic level, in accordance to the observed tendency, although not significant after multiple correction, towards the reduction of the expression of additional pro-inflammatory genes such as *JAK1 (p=0.018), PATFR (p=0.013), CXCR1 (p=0.01), GPD2*, and glycolytic genes *LDHA (p=0.003) and IRS1(p=0.026)*, which have been associated to immunosuppression in cancer (46–49) (**Supplementary Table 4**).

## 4 Discussion

Lung cancer is one of the most deadly and common types of cancer in the world (1). Although the identification of targetable molecular pathways such as EGFR inhibitors has improved the treatment and outcome of patients, majority of NSCLC tumours develop drug resistance and new generation of targeted therapeutic drugs, alone and/or in combination with different drugs, are being developed.

Nutrients can directly affect fundamental cellular processes and some diet derived ingredients, bioactive natural compounds and natural extracts have been shown to inhibit the tumour growth in preclinical and clinical trials (15).

We have previously described a supercritical extract of rosemary (*Rosmarinus Officinalis*) SFRE (EFSA approved for human use) as a potential antitumor agent in colon and breast cancers by mean of the inhibition of lipid metabolism related targets and DNA synthesis, synergising with 5-FluoroUracil (5-FU) in the inhibition of cell proliferation both *in vitro* and in preclinical models (16–19).

Herein, we aimed to investigate the potential antitumor effects of SFRE to be proposed as a co-adjuvant in the treatment of NSCLC.

*In vitro* treatment of NSCLC cells, H1299 and H1975, indicates that SFRE inhibits cell proliferation in a dose dependent manner (**Figure 1**). Importantly, SFRE diminishes the two main bioenergetic pathways, aerobic glycolysis (**Figure 2**) and mitochondrial oxidative phosphorylation (**Figure 3**), compromising the intracellular levels of ATP of tumour cells and activating apoptosis (**Supplementary Figure 1**).

Altered lipid metabolism has been found in many types of cancer and, consequently, lipid metabolism genes could constitute a prognostic and therapeutic tool in different tumours (31). Rosemary extracts has been described to inhibit relevant molecular pathways and transcription factors implicated in lipid metabolism (50, 51).

In a previous work, SFRE diminished lipid metabolism in CRC, and for this reason we hypothesized SFRE may affect lipid metabolism in NSCLC (25).

The analysis of lipid metabolism targets related to the *de novo* lipogenesis (*SREBF1, FASN, SCD*1) and cholesterogenesis (*HMGCR*), activation of fatty acids (*ACSL*s), homeostasis of cholesterol (*ABCA1, ApoA1*), biosynthesis and remodelling of membrane phospholipids (*CHKA, AGPAT*), as well as lipid related oncogenic pathways implicated in cell proliferation (*EGFR*) and inflammation (*JAK1*), indicated a relevant role of SFRE on the inhibition of lipid metabolism reprogramming in NSCLC cells. These effects are in line with the reduction of the intracellular neutral lipid content as well as main phospholipids, indicating that the inhibition of lipid metabolism may be on the bases of the inhibition of the cell proliferation and/or cell survival pathways (**Figures 4**, **5**).

Fatty acids (FAs) are indispensable components of cellular membranes, and they are essential for posttranslational protein modifications; moreover, they are energy generators and contribute to maintain redox homeostasis through β-oxidation. *De novo* lipogenesis is considered a new hallmark in many aggressive cancers. Overexpression of Fatty Acid Synthase (*FASN*) correlates with poor prognoses and treatment resistance in NSCLC (10), and stearoyl CoA desaturase 1 (*SCD1*) is highly expressed in lung adenocarcinomas promoting *in vitro* and *in vivo* tumorigenesis, cell migration and invasion (52). Acyl-coenzyme A synthetase long chain family members (ACSLs) are key components for the control of lipid synthesis and β-oxidation. ACSLs suppression is associated with depletion of cellular ATP causing the death of lung cancer cells (53–55). Other fundamental structural component of lipid membranes is cholesterol, that is also essential for cellular proliferation and tumour microenvironment remodelling (21). Tumour cells show a powerful fatty acid and cholesterol avidity which, together with the increased lipogenesis and cholesterogenesis (*HMGCR*), can be partially satisfied by increasing the uptake of exogenous lipids and/or lipoproteins (9). Increased serum cholesterol levels have also been associated with higher risk of cancer and poorer prognosis (22). ABCA1 contributes to maintain the cellular cholesterol homeostasis through the transfer of phospholipids and cholesterol to apolipoprotein A1 (*ApoA1*). In cancer cells, ABCA1 has been associated multidrug resistance as a drug efflux transporter and it is a key component of the tumour microenvironment remodelling. Related to ABCA1 come into sight the biosynthetic process of cholesterol. HMG-CoA reductase (*HMGCR*) is highly regulated and represents the rate-limiting step. Interestingly, HMGCR is the target for cholesterol lowering drugs known as statins (56, 57).

The successful application of bioactive compounds as co-adjuvants requires the demonstration of no interference and/or positive synergism with the therapeutic treatments in the clinic. For this reason, we next evaluated the effects of SFRE in combination with main standard drugs such as cisplatin, pemetrexed and pembrolizumab, used in the clinic in NSCLC patients.

Cisplatin is one of the most common first-line treatments in NSCLC, excluding targeted therapies. Importantly, the combination index (CI) after treatment NSCLC cell lines with SFRE for three hours at a fixed dose of 1/2 IC_50_ for H1299 and H1975, indicated a positive synergism between cisplatin and SFRE (**Figure 7A**). The analysis of the expression of these genes in NSCLC cells after the treatment with SFRE indicated the downregulation of *TK1* and *TYMS* (**Figure 7B**) which may explain, at least partially, the observed synergism between SFRE and cisplatin in the inhibition of cell proliferation.

During the last decade, the place of pemetrexed for the treatment of non-squamous NSCLC became established. Pemetrexed, an antifolate agent, is one of the recommended drugs combined with cisplatin or carboplatin for first-line treatment of these patients. Pemetrexed inhibits cell replication and growth by reducing the expression of three enzymes involved in purine and pyrimidine synthesis: thymidylate synthase (*TYMS*), dihydrofolate reductase (*DHFR*) and glycinamide ribonucleotide formyl-transferase (*GARFT*) (37). Importantly, the combination index (CI) after the treatment of H1299 and H1975 with SFRE for three hours at a fixed dose of 1/2 IC_50_ indicated a positive synergism between pemetrexed and SFRE (**Figure 8A**). The analysis of the expression of dihydrofolate reductase (*DHFR*) and glycinamide ribonucleotide formyl-transferase (*GARFT*) genes in NSCLC cells after the treatment with SFRE indicated the downregulation of both genes (**Figure 8B**), which may explain, at least partially, the observed synergism between SFRE and pemetrexed in the inhibition of cell proliferation.

Immunotherapy has made the breakthrough in cancer therapeutics by mean of immune regulatory mechanisms to enhance the immune response against cancer cells. Clinical studies evaluating the response to immunotherapy in lung cancer have demonstrated superior survival indices as well as reduced toxicity profiles in comparison to standard chemotherapy regimens. Of note, anti-PD-1 therapy (pembrolizumab) has recently replaced chemotherapy in the first line treatment for NSCLC with high PD-L1 expression, and the addition of pembrolizumab to platinum chemotherapy resulted in a significant improvement in overall survival (OS) in patients with non-squamous NSCLC, regardless of PD-L1 expression. For this reason, we aimed to evaluate if SFRE could act synergistically with pembrolizumab in the inhibition of the cell proliferation of NSCLC cells. A shown in **Figure 9A**, the combination index (CI) indicated a positive synergism between pemetrexed and SFRE in the inhibition of NSCLC cell proliferation. In addition, SFRE diminished the expression of PD-L1 and CX3CR1 which exert immunosuppressive effects at the tumour microenvironment (45), (**Figure 9C**) suggesting SFRE may contribute to diminish immune evasion of the lung cancer cells.

Due to the observed effects of SFRE in the inhibition of lipid metabolism targets in NSCLC cells together with its synergism with therapeutic drugs used in the treatment of NSCLC patients, next, we wanted to evaluate the relevance of SFRE in the clinic as a putative co-adjuvant in the treatment of NSCLC patients.

With this objective, and in collaboration with the Medical Oncology Service of Infanta Sofia University Hospital, we evaluated the effects of SFRE on the expression of a selected panel of genes related to lipid metabolism, inflammation, oxidative stress, immune system, and oncogenic pathways in PBMC from NSCLC patients.

The evolution of gene expression in PBMCs was compared from the initial visit (V1) to visit 4 (nine weeks of intervention) to visit 6 (sixteen weeks of intervention) at the two treatment groups. The mixed model analysis of gene expression (ΔCt) for the evolution of gene expression across time for the two groups, treatment (CR) vs control (CC), showed statistically significant visit x treatment interactions after multiple test corrections for *NLRP3, MAPK, SREBF1*Other genes, such as *JAK1, LDHA, PATFR, CXCR1, GPD2* and *IRS1* showed statistically significant interactions, although significance was lost after multiple test correction (**Supplementary Table 4**).The differential time evolution observed for *NLRP3, MAPK, SREBF1* is of gene expression inhibition in the case of the CR group, and slight exacerbation in the case of the CC group (**Figure 10**).

These results suggest that the CR group displays a better control of lipid metabolism-related inflammatory pathways, in agreement also with the observed trend for reduction of expression in additional pro-inflammatory genes like *JAK1, PATRF, CXRC1, GPD2*, and glycolytic genes *LDHA* and *IRS1*, which are associated to immunosuppression in cancer (46–49).

The observed effects of SFRE on PBMCs lipid metabolism may be relevant in the management of NSCLC in the clinic. By one hand, increased lipid metabolism may lead to an exacerbated inflammation leading to the recruitment of immunosuppressive cells, such as bone marrow myeloid derived immunosuppressive cells and/or regulatory T cells (Treg) at the local tumour microenvironment. On the other hand, abnormal lipid accumulation in PBMCs subpopulations related to the innate immunity, such as dendritic cells (DC), have been shown to compromise the immune response at the local tumour microenvironment in patients with lung cancer (58) by reducing their antigen handling capacity, downregulating co-stimulating molecules such as CD86, and/or overexpressing tolerogenic cytokine IL-10 (59). Targeting FASN upregulation of the tumour-promoting pathway can enhance anti-tumour immunity (60, 61). In addition, lipid metabolism mediators such as inflammasome and prostaglandins are also associated to the production of lactate at the tumour microenvironment with immunosuppressive effects. Moreover, lactate has been proposed as a prognostic and predictive biomarker in several types of cancer (62).

In summary, SFRE exerts antitumour effects in NSCLC cells by diminishing lipid metabolism in cancer cells. In addition*, in vitro* experiments indicate that SFRE synergises with therapeutic drugs used in the clinic, such as cisplatin, pemetrexed and pembrolizumab. Finally, the clinical relevance of SFRE in NSCLC patients is suggested in a pilot intervention study where SFRE formulated with bioactive lipids (PCT/ES2017/070263) diminishes lipid metabolic and inflammatory targets in PBMC which have been shown to diminish the immune system antitumour functions. Based on these results, SFRE can be proposed as a co-adjuvant in the treatment of NSCLC that merits further investigation.

Main limitations of the study are the small number of patients analysed, heterogeneity in the clinical characteristics, including treatments and nutritional status, and the analysis of gene expression in PBMCs without the identification of the individual subpopulations responsible of the observed changes in gene expression.

## Data availability statement

The original contributions presented in the study are included in the article/**Supplementary Material**. Further inquiries can be directed to the corresponding authors.

## Ethics statement

The study was conducted according to the guidelines of the Declaration of Helsinki and approved by the Ethics Committee for Clinical Investigation of La Paz University Hospital (Ref. HULP 5617). The patients/participants provided their written informed consent to participate in this study.

## Author contributions

Conceptualization, MGC, GR and ARM. Methodology, AB, MGC, JML-L.; formal analysis, AB, MGC, GC, BT and JML-L. Investigation, JM, JM-R, AB, MGC. and JML-L.; writing—original draft preparation, AB and MGC writing—review and editing, AB, MGC, GR and ARM. Clinical trial and data of patients: MS, EC and ARM supervision, MGC, GR and AM. Funding acquisition, GR and ARM. All authors contributed to the article and approved the submitted version

## Funding

Funding: This research was funded by Regional Government of Community of Madrid (IND2017/BIO-7857; P2018/BAA-4343-ALIBIRD2020-CM), Ministerio de Ciencia e Innovación, Spain (PID2019-110183RB-C21); Ramon Areces Foundation (CIVP19A5937); EU Structural Funds and COST Action (CA17118); Synergistic Projects Community of Madrid (NUTRISION-CM/Y2020/BIO-6350) and REACT EU Program (Comunidad de Madrid and The European Regional Development Fund. ERDF. European Union- FACINGLCOVID-CM project). Adrián Bouzas has a predoctoral grant from the industrial predoctoral program of Community of Madrid (IND2017/BIO-7857).

## Conflict of interest

The authors declare that the research was conducted in the absence of any commercial or financial relationships that could be construed as a potential conflict of interest.

## Publisher’s note

All claims expressed in this article are solely those of the authors and do not necessarily represent those of their affiliated organizations, or those of the publisher, the editors and the reviewers. Any product that may be evaluated in this article, or claim that may be made by its manufacturer, is not guaranteed or endorsed by the publisher.
