# Supplementary material for: Phenolic diterpenes from Rosemary supercritical extract inhibit non-small cell lung cancer lipid metabolism and synergise with therapeutic drugs in the clinic
Source: Front Oncol. 2022 Nov 9;12:1046369. doi: 10.3389/fonc.2022.1046369 (PMC9682134; doi:10.3389/fonc.2022.1046369)
Supplement: Supplementary file 1 [file DataSheet_1.zip › Supplementary Table 1 Oligos & Probes.docx]

**List of oligos and Taqman probes used in this study.**

***SREBF1*** F-CCGCCGCGCCTTGAC; R-AGCATAGGGTGGGTCAAATAGG

***FASN*** F-TATGAAGCCATCGTGGACGG; R-GAAGAAGGAGAGCCGGTTGG

***SCD-1*** F-TGCCCACCACAAGTTTTCAG; R-CATCAGCAAGCCAGGTTTGT

***HMGCR*** F-TGATTGACCTTTCCAGAGCAAG; R-CTAAAATTGCCATTCCACGAGC

***APOA1*** F-CCCTGGGATCGAGTGAAGGA; R-CTGGGACACATAGTCTCTGCC

***ABCA1*** F-ACCCACCCTATGAACAACATGA; R-GAGTCGGGTAACGGAAACAGG

***LDLR*** F-CAGATATCATCAACGAAGC; R-CCTCTCACACCAGTTCACTCC

***AGPAT1*** F-CCCCCACCATTCCTACCGC; R-CATGCCCCTGGCCACAAATC

***ACSL1*** F-TTGGGAAGGATTCTGGTCTG; R-TGCCATTTCCTCTG GCTTT

***ACSL3*** F-GGCGTAGCGGTTTTGACAC; R- CCAGTCCTTCCCAACAACGA

***ACSL4*** F-GGCACAACAGAAAGGGGTAG; R-GGTTCCTCAGCTCCTTCCTT

***CASP9*** F-CAGGCCCCATATGATCGAGG; R-TCGACAACTTTGCTGCTTGC

***CHOP*** F- GGAGAACCAGGAAACGGAAAC; R-TCTCCTTCATGCGCTGCTTT

***DHFR*** F-GCTGCTGTCATGGTTGGTTC; R-GAGGTTGTGGTCATTCTCTGGA

***GARFT*** F-CTGGAAAAGGGGTGATTGTTGC; R-TCAGTGAAACACAGACACGAC

***PDL1*** F-GGTGCCGACTACAAGCGAAT; R-GGTGACTGGATCCACAACCAA

***GLUT4*** F- ATCCTTGGACGATTCCTCATTGG; R- CAGGTGAGTGGGAGCAATCT

***GAPDH*** F-TGGTATCGTGGAAGGACTCATGAC; R-ATGCCAGTGAGCTTCCCGTTCAGC

***B2M*** F-GATGAGTATGCCTGCCTGCCGTGT; R-TGCGGCATCTTCAAACCTCC

***ALDH1A1*** F-TGTTAGCTGATGCCGATCTG; R-TTCTTAGCCCGCTCAACACT

***CD29*** F-CATCTGCGAGTGTGGTGTCT; R-GGGGTAATTTGTCCCGACTT

***CD44*** F-AGCAGCGGCTCCTCCAGTGA; R-CCCACTGGGGTGGAATGTGTCT

***EpCAM*** F-CGCAGCTCAGGAAGAATGTG; R-TGAAGTACACTGGCATTGACG

***E cadh*** F-GAACGCATTGCCACATACAC; R-GAATTCGGGCTTGTTGTCAT

***K18*** F-GAGTATGAGGCCCTGCTGAA; R-CAGACACCACTTTGCCATCC

***Na K ATPase*** F-GCCTCCCAAGAATGAGTCCT; R-ATTTGGGCTGCAGGAGTTTG

***N cadh*** F-CGGTTTCATTTGAGGGCACA; R-TTGGAGCCTGAGACACGATT

***Vim*** F-GAGTCCACTGAGTACCGGAG; R-ACGAGCCATTTCCTCCTTCA

***Taqman probes:*** *CHKA- Hs00957878_m1; BMP2- Hs00154192_m1; NEF2L2- Hs00232352_m1; JAK1- Hs01026983_m1; TYMS - Hs00426591_m1; TK1 - Hs01062125_m1; B2M Hs00187842_m1; GAPDH Hs99999905_m1*
