# Supplementary figures and images for: Phenolic diterpenes from Rosemary supercritical extract inhibit non-small cell lung cancer lipid metabolism and synergise with therapeutic drugs in the clinic
Source: Front Oncol. 2022 Nov 9;12:1046369. doi: 10.3389/fonc.2022.1046369 (PMC9682134; doi:10.3389/fonc.2022.1046369)

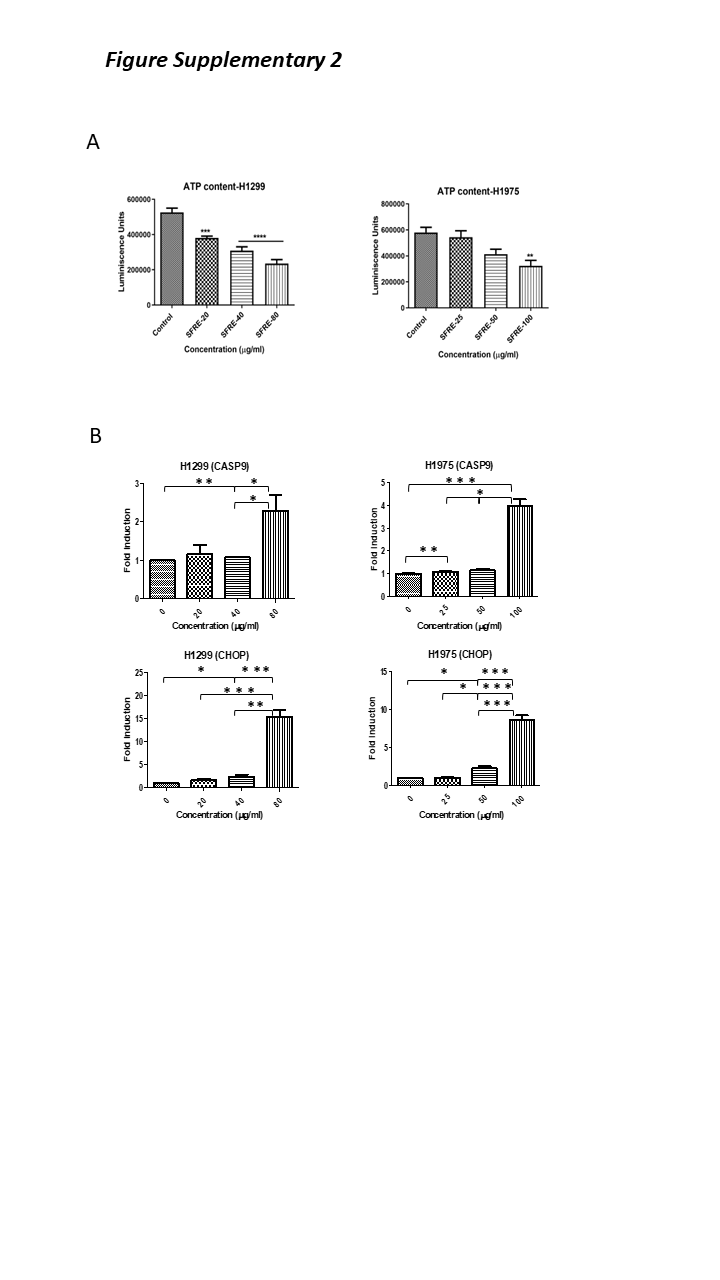

Supplement: Supplementary file 1 [file DataSheet_1.zip › Supplementary Fig 2 FiN.tif]

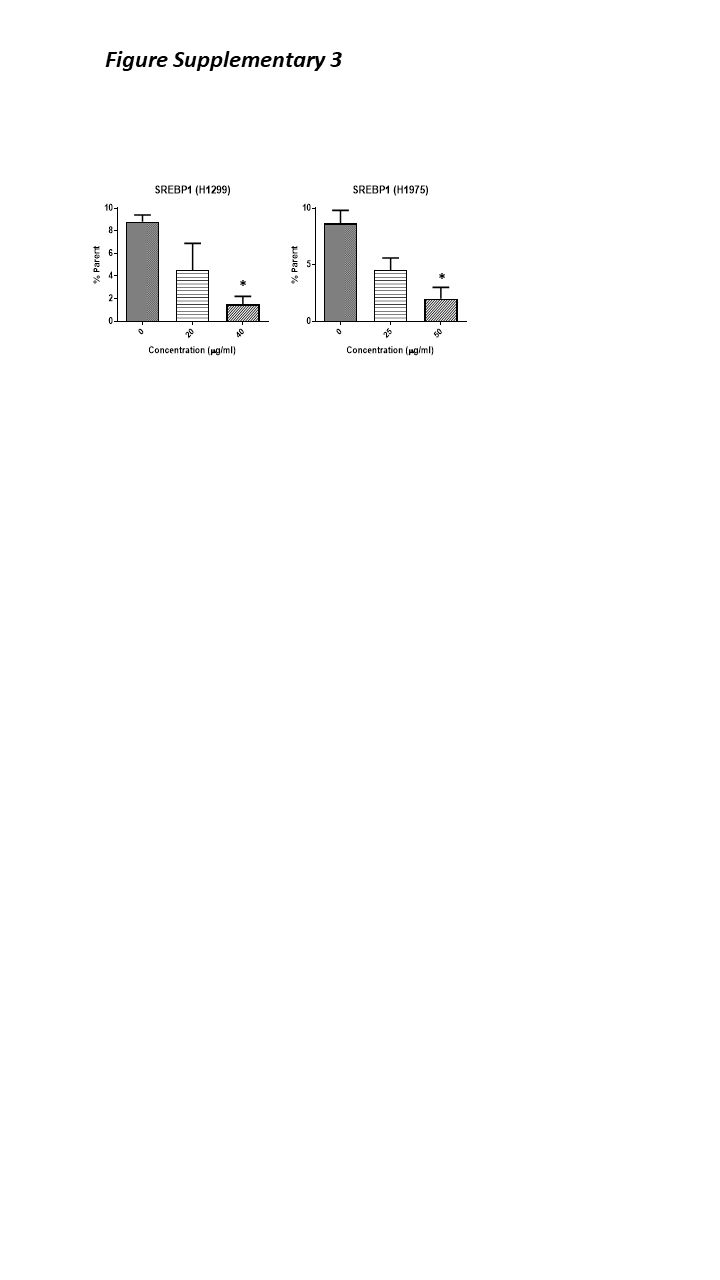

Supplement: Supplementary file 1 [file DataSheet_1.zip › Supplementary Fig 3 FiN.tif]

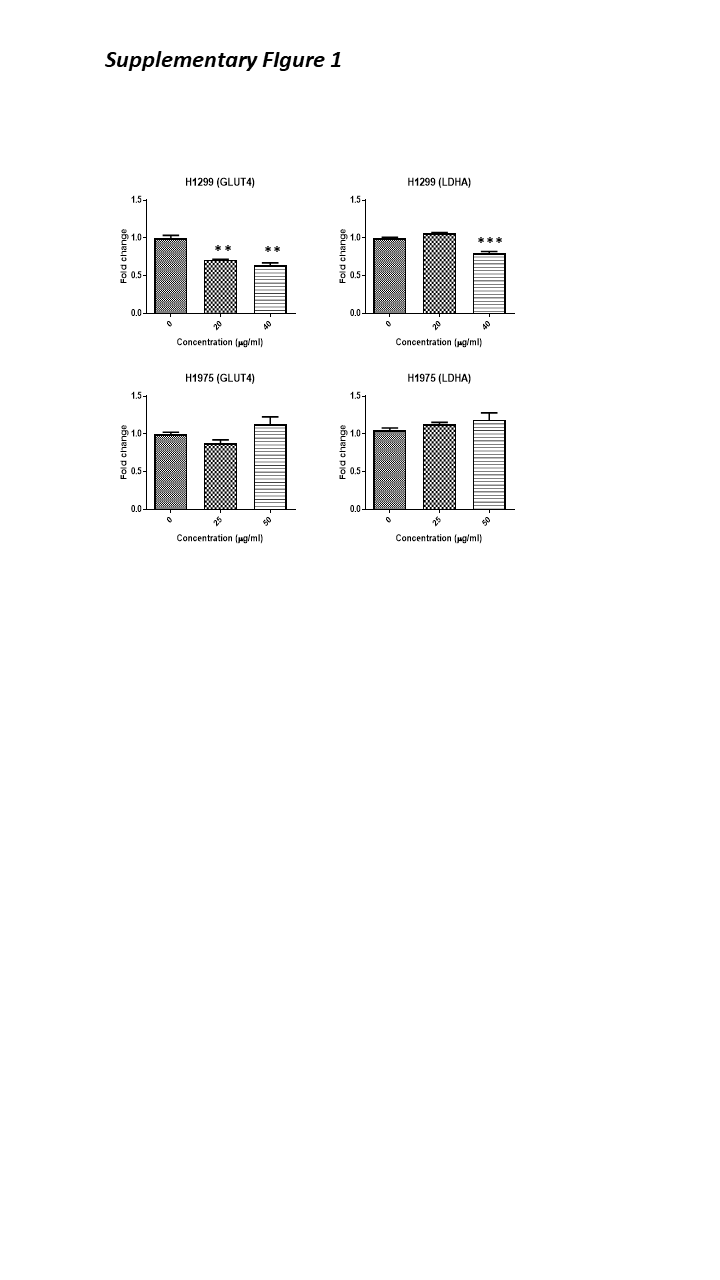

Supplement: Supplementary file 1 [file DataSheet_1.zip › Supplementary Fig1 FiN.tif]
